# A shallow scattering layer structures the energy seascape of an open ocean predator

**DOI:** 10.1126/sciadv.adi8200

**Published:** 2023-10-04

**Authors:** Martin C. Arostegui, Barbara Muhling, Emmett Culhane, Heidi Dewar, Stephanie S. Koch, Camrin D. Braun

**Affiliations:** ^1^ Biology Department, Woods Hole Oceanographic Institution, Woods Hole, MA, USA.; ^2^ Institute of Marine Sciences, University of California, Santa Cruz, Santa Cruz, CA, USA.; ^3^ Fisheries Resources Division, Southwest Fisheries Science Center, National Marine Fisheries Service, National Oceanic and Atmospheric Administration, La Jolla, CA, USA.; ^4^ Department of Biological Sciences, Thomas More University, Crestview Hills, KY, USA.

## Abstract

Large predators frequent the open ocean where subsurface light drives visually based trophic interactions. However, we lack knowledge on how predators achieve energy balance in the unproductive open ocean where prey biomass is minimal in well-lit surface waters but high in dim midwaters in the form of scattering layers. We use an interdisciplinary approach to assess how the bioenergetics of scattering layer forays by a model predator vary across biomes. We show that the mean metabolic cost rate of daytime deep foraging dives to scattering layers decreases as much as 26% from coastal to pelagic biomes. The more favorable energetics offshore are enabled by the addition of a shallow scattering layer that, if not present, would otherwise necessitate costlier dives to deeper layers. The unprecedented importance of this shallow scattering layer challenges assumptions that the globally ubiquitous primary deep scattering layer constitutes the only mesopelagic resource regularly targeted by apex predators.

## Teaser

A shallow scattering layer appears vital to achieving bioenergetic balance for pelagic predators foraging in oligotrophic waters.

## INTRODUCTION

In the deep open ocean, the greater relative productivity of pelagic than benthic energy pathways favors the dominance of large pelagic predatory fishes over demersal counterparts (*1*, *2*). These pelagic predators exhibit the highest metabolic demand known among marine fishes as a result of intense selection for the capacity to actively and visually pursue mobile prey in offshore waters (*3*), in some cases yielding the phenotypic extreme of endothermy (*4*). The foraging environment of highly migratory pelagic predators constitutes a dynamic energy seascape of movement costs and benefits that shift across space and time to determine energetic profitability; this, in turn, structures predator selection of habitats and their directed movements among them (*5*). Notably, predators with high energy expenditure typically target prey with greater energetic content (*6*) and feed in areas with minimized energetic costs (*7*), thereby increasing net energetic profit. The similarity in movement patterns across diverse predator lineages, as well as their change from coastal to open ocean regions (*8*), is consistent with altering foraging strategies in response to a variable energy seascape (*9*). Coastal regions exhibit seasonal peaks in primary productivity that can lead to predator occurrence tracking temporary resource waves (*10*). In comparison, while much of the open ocean is consistently characterized by low prey biomass in well-lit near-surface waters during the day but high prey biomass in dim midwaters (*11*), distributional characteristics of deep resources govern whether large pelagic predators surpass the threshold of energetic viability in unproductive waters (*12*).

The ocean twilight zone harbors the greatest biomass of animals on the planet, the fishes alone of which are estimated to constitute in excess of 11 billion (metric) tons (*13*). Some of these organisms form dense aggregations that are readily detectable with scientific echosounders as acoustic scattering layers (*14*). The vertical distributions of these layers are bounded within distinct ranges of light intensities that govern tradeoffs in predation risk and foraging opportunity (*15*). The largest and most researched is the primary deep scattering layer (DSL) prevalent throughout the world ocean at a mean depth of ∼500 m and covering a vertical extent of >200 m (*16*, *17*). While the daytime occurrence of a single DSL is commonly observed, multiple scattering layers comprising different communities may be present and vary in number across a range of spatial and temporal scales (*18*, *19*, *20*). Depending on the region in the ocean, ∼20 to 95% of scattering layer organisms may undergo diel vertical migration in which they occupy mesopelagic depths during the day and epipelagic depths at night; the remaining portion is nonmigrant and maintains a largely constant mesopelagic depth distribution across the diel cycle (*16*).

There is increasingly more documentation of surface–to–deep ocean connectivity driven by pelagic predators foraging on abundant scattering layer prey or other organisms associated with these layers (*21*). For example, oceanographic features that harbor more scattering layer biomass (*22*) aggregate diverse pelagic predators (*23*), highlighting that environmental variability influences the trophic connectivity between predators and scattering layer prey (*24*). This connectivity between scattering layers and predators is complex, as oceanography affects both the prey and predators. Light penetration is a major determinant of scattering layer depth (*25*), while other extrinsic factors, such as temperature or oxygen, interact with physiological constraints to determine what depths predators may occupy and, thus, which specific layer(s) they may target (*26*). However, we lack knowledge on how predators achieve energy balance in the open ocean, including the cost of deep diving and changes in metabolic rate while targeting scattering layers across biomes with varying light and physiologically relevant conditions at depth. Accounting for environmental influence on metabolic rate is necessary to understand the bioenergetic tradeoffs that structure predator behavior and trophic linkages to prey (*27*). Given that vast swaths of the open ocean have long been considered a marine desert (*28*), these bioenergetic considerations are crucial to understanding the seasonal, age-specific, or lifelong persistence of predators in seemingly nutrient-poor biomes.

Here, we use data from archival tags deployed on a model pelagic predator, albacore tuna (*Thunnus alalunga*), to assess how the bioenergetics of scattering layer forays vary across North Pacific biomes. Tag-measured depth and relative light levels permit us to reconstruct the subsurface light attenuation environment experienced by the predators, including increased attenuation putatively caused by chlorophyll maxima in the euphotic zone and scattering layers below. With a bioenergetics model of total metabolic rate as a function of vertical swimming speed and in situ water temperature, we estimate the energetic cost of deep diving to scattering layers across oligotrophic to mesotrophic and subtropical to temperate waters. In concert, these analyses enable us to assess how the specific scattering layer that a thermally limited predator targets may transition across oceanographic regimes with differing energy seascapes.

## RESULTS

### Migration

**Fig. 1.**
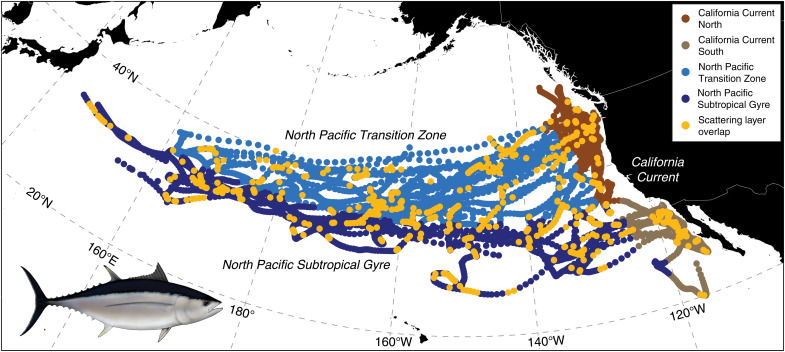
Distribution and scattering layer overlap of North Pacific albacore. Distribution of tagged juvenile albacore (*n* = 25) across North Pacific biomes, including locations of putative overlaps with scattering layers during daytime dives below the euphotic zone. The map is shown on the Lambert Conformal Conic projection of the North Pacific.

Archival tags were deployed on juvenile albacore (*n* = 1086 individuals) in the Northeast Pacific that logged high-resolution depth, temperature, and relative light data for each individual for up to nearly 3 years. We recovered 25 of these tags that recorded 10,360 deployment days spanning >235,000 km of cumulative horizontal travel, primarily across the California Current, North Pacific Transition Zone, and North Pacific Subtropical Gyre (Fig. 1 and table S1). Most individuals tagged in the northern California Current migrated seasonally, moving westward along the North Pacific Transition Zone in fall and returning to the California Current in late spring or early summer (fig. S1A). A subset of albacore tagged in the southern California Current moved offshore into the North Pacific Subtropical Gyre, but these fish migrated shorter distances and with less predictable seasonality than those tagged in the northern California Current. Other individuals tagged in the southern California Current remained resident in this area for the entirety of their time at large, up to 343 days. Albacore do extensive diving throughout these North Pacific biomes, with daily cumulative vertical travel throughout the water column averaging 7.9 km in the northern California Current, 9.4 to 9.6 km in the southern California Current and North Pacific Transition Zone, and 12.2 km in the North Pacific Subtropical Gyre. The quantity of vertical movement generally increased in concert with subsurface temperature and light across these biomes, with albacore experiencing colder and darker conditions in the California Current than in the North Pacific Subtropical Gyre and Transition Zone over the depth range of the lower epipelagic and upper mesopelagic (fig. S1B).

### Energetics

**Fig. 2.**
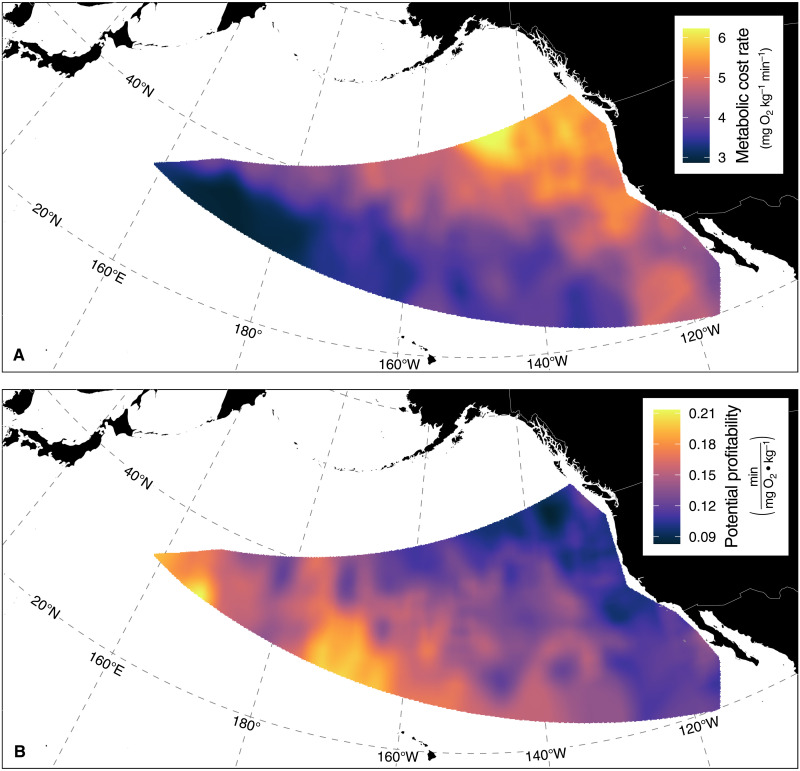
Energy seascapes of daytime deep foraging dives. The energy seascapes of daytime deep foraging dives below the euphotic zone in terms of the metabolic cost rate (**A**) and potential profitability (**B**). Spatial fields were generated with inverse distance weighted interpolation. The euphotic zone here is defined as the portion of the water column shallower than the lower limit (99.7th percentile) of deep chlorophyll maxima (DCM) depth distribution per 10° latitudinal band, as determined in a global analysis of >500 biogeochemical Argo profiling floats yielding >68,000 vertical chlorophyll profiles (*70*).

Idealized deep dives consistent with sustained foraging (U-shaped) and prey searching (V-shaped) below the euphotic zone revealed behavioral variation among oceanographic regimes. Foraging dives (*n* = 2856) were characterized by a high proportion of bottom time at a target depth and longer overall dive duration that averaged ∼20 min (range, 10 min to >2 hours). Searching dives (*n* = 10,338) were characterized by a low proportion of bottom time and shorter average dive duration of 8 min (range, 5 to 23 min). We restricted our analyses to daytime deep dives because such behavior almost never occurs at night when these fish are distributed nearly exclusively in the upper epipelagic. Across dive types and biomes, a power function relating the metabolic cost rate to the minimum ambient temperature experienced during a deep dive revealed that for every 10% increase in the minimum ambient temperature, there was a linear approximation of an 8% decrease in the metabolic cost rate. The mean temperature conditions and corresponding metabolic and behavioral outcomes experienced by albacore while conducting daytime dives below the euphotic zone largely varied along a northeast to southwest gradient (Fig. 2). The shallowest (mean, 29 m) and coldest (mean, 15.5°C) mixed layer, the lowest minimum temperature at depth (mean, 7.4°C), and the largest change in ambient temperature (mean, −8.6°C) experienced during such dives occurred in the northern California Current, which corresponded to the highest metabolic cost rate in this study (mean, 5.7 mg O_2_ kg^−1^ min^−1^ for U dives; fig. S2; see table S2 for all U and V dive metrics). The high metabolic cost rate in this region was consistent with the highest dive descent rate (mean, 1.0 m s^−1^) and the shortest foraging dive duration (mean, 16 min) and bottom time (mean, 10 min). In contrast, albacore conducting foraging dives in the North Pacific Subtropical Gyre experienced the deepest (mean, 55 m) and warmest (mean, 17.0°C) mixed layer, the warmest minimum temperature at depth (mean, 10.7°C), and the smallest change in ambient temperature (mean, −6.2°C), driving the lowest metabolic cost rate (mean, 4.2 mg O_2_ kg^−1^ min^−1^) and dive descent rate (mean, 0.7 m s^−1^). This 26% decrease in the mean metabolic cost rate of a deep foraging dive in the North Pacific Subtropical Gyre relative to the northern California Current was associated with a longer foraging dive duration (mean, 21 min) and bottom time (mean, 13 min) as albacore transitioned from colder to warmer biomes (fig. S2). Similarly, a proxy for the potential profitability of a deep foraging dive, defined as the bottom time (or available foraging time) per unit metabolic cost, increased 37% from the northern California Current (mean, 0.103 min mg O_2_^−1^ kg) to the North Pacific Subtropical Gyre (mean, 0.141 min mg O_2_^−1^ kg).

### Light attenuation

**Fig. 3.**
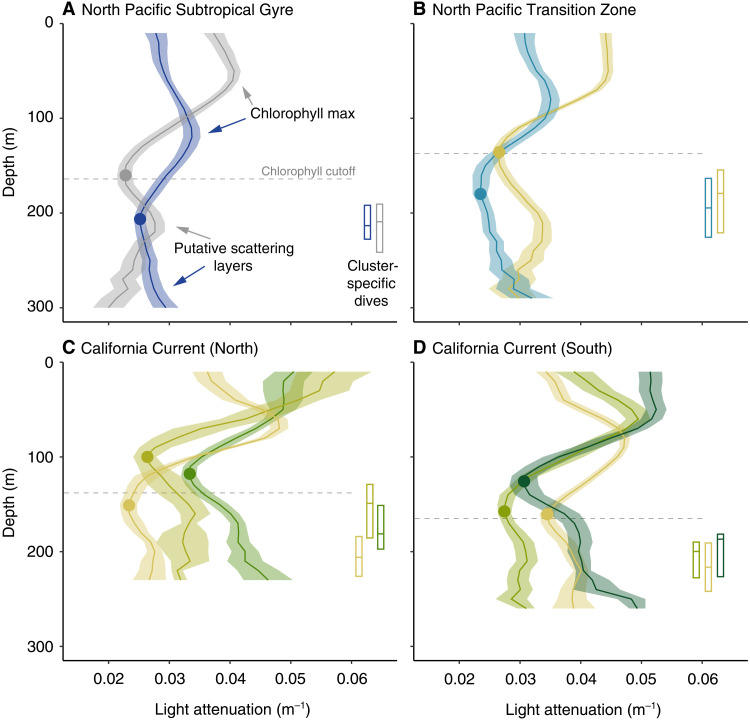
Light attenuation profiles with scattering layer overlap. Composite profiles of light attenuation clusters within each biome (**A** to **D**) that included deep increases in attenuation consistent with scattering layer overlap (mean ± 95% confidence interval). Profiles are color-scaled from low (blue), intermediate (white), to high (green) mean attenuation over depths ≤100 m. The cluster-specific median (and interquartile range) of the maximum depth reached during dives potentially representing sustained foraging or prey searching behaviors are indicated by color-matched box plots. The depth cutoffs below which chlorophyll should not contribute to light attenuation are indicated by the dashed horizontal lines; these climatological values come from the latitudinal band primarily encompassing the dive data within each biome (see the “Light attenuation profiling” section in Methods for details). The within-profile attenuation minima against which deep increases in attenuation (below the chlorophyll cutoff) were assessed for scattering layer overlap are indicated by the filled circle. Examples of increased attenuation driven by chlorophyll at shallower depths and putative scattering layers at deeper depths are highlighted in (A).

Subsurface relative light levels recorded by the tags quantified the midday (local solar noon ± 1 hour) light attenuation environment and suggested diving albacore overlap with putative scattering layers below the euphotic zone across their entire North Pacific range (Fig. 1). Light attenuation was highest in epipelagic waters (calculated from 20 to 150 m for all regions) of the mesotrophic northern California Current (mean, 0.041 m^−1^) and lowest in the oligotrophic North Pacific Subtropical Gyre (mean, 0.035 m^−1^); these conditions were reflected in the decrease in light experienced by albacore during deep foraging dives, which was largest in the northern California Current (mean, −4.13 orders of magnitude) and smallest in the North Pacific Subtropical Gyre (mean, −3.39 orders of magnitude). Colocating light attenuation profiles to climatological deep chlorophyll maxima (DCM) depth by latitude allowed us to isolate peaks in attenuation well below DCM depth (>3 SDs below the mean; Fig. 3). Of the deployment days with light attenuation profiles passing quality control and extending below the DCM depth threshold (*n* = 3968), 17% exhibited increases in attenuation at depths suggestive of scattering layers. This presence-only detection method was designed to conservatively identify putative overlap with scattering layers only where chlorophyll could not be confounding the attenuation signal. Using a within-biome clustering approach of attenuation profiles with putative scattering layer overlap, we identify important features governing the attenuation of light within and across depth strata. For example, high surface productivity in the California Current leads to at least some profiles with high attenuation in the top 50 m (Fig. 3, C and D). Similarly, DCM are apparent in at least one profile cluster within each biome and drive peaks in attenuation centered at depths up to 100 to 140 m in the North Pacific Subtropical Gyre, 60 to 100 m in the North Pacific Transition Zone, 50 to 90 m in the northern California Current, and 60 to 100 m in the southern California Current (Fig. 3). In contrast, peaks in attenuation presumably driven by scattering layers occur below ∼200 m in the North Pacific Subtropical Gyre and Transition Zone, 150 m in the northern California Current, and 180 m in the Southern California Current (Fig. 3). Furthermore, as the observed depths of putative scattering layers deepened within and across biomes so did depths of albacore foraging and searching dives (Fig. 3).

### Scattering layers

**Fig. 4.**
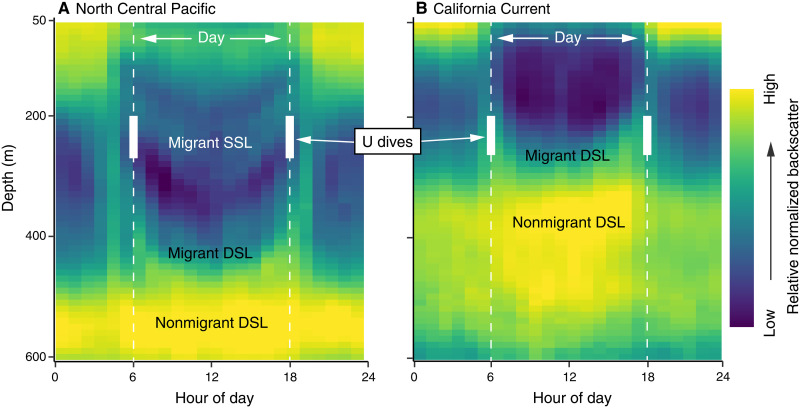
Bioacoustic composites of scattering layers. Acoustic Doppler current profiler (ADCP) composites of scattering layer vertical distribution over the 24-hour diel cycle in the oligotrophic North Central Pacific (**A**) and mesotrophic California Current (**B**). Data from the North Pacific Subtropical Gyre and Southern Transition Zone jointly constitute the composite of the North Central Pacific (A), a mesopelagic ecoregion distinct from that of the California Current (*46*). Data from the California Current are of the southern region (B), the composite of which is parallel to that of the northern region and representative of the biome as a whole. Daytime (hours 6 to 18) and nighttime (hours 0 to 6 and 18 to 24) are separated by the white dashed vertical lines, with the ecoregion-specific interquartile range of maximum depths reached during daytime deep foraging (U) dives bounded by the solid sections.

Diel composites of shipboard acoustic measurements from 0 to 600 m highlighted biome-level variation in scattering layer depth and structure (Fig. 4). The high attenuation environment of the California Current was characterized by two primary scattering layers in the northern and southern regions: (i) a migrant DSL distributed below ∼200 to 220 m at local noon but ascending to <100 m at night and (ii) a nonmigrant DSL centered at ∼400 m across diel periods (Fig. 4B). Accordingly, maximum foraging dive depth in the northern (mean, 175 m) and southern (mean, 220 m) California Current and concurrent increased light attenuation at depth (Fig. 3, C and D) were consistent with overlap of the migrant DSL at ∼200 m but not the nonmigrant DSL that occurs markedly deeper (∼400 to 500 m; Fig. 4B). In contrast, three distinct mesopelagic scattering layers were apparent in the North Central Pacific (i.e., across the North Pacific Subtropical Gyre and Southern Transition Zone): (i) a migrant shallow scattering layer (SSL) distributed from ∼200 to 300 m at local noon but ascending to <200 m at night, (ii) a migrant DSL distributed below ∼400 m at local noon but ascending to <200 m at night, and (iii) a nonmigrant DSL centered at ∼550 m that was stationary across the diel cycle (Fig. 4B). Maximum depths reached by albacore conducting daytime foraging dives below the euphotic zone in the North Pacific Transition Zone (mean, 208 m) and North Pacific Subtropical Gyre (mean, 218 m) were consistent with overlap of the migrant SSL but not the migrant nor nonmigrant DSLs below (Fig. 4A). Furthermore, concurrent increased light attenuation at depth in these North Central Pacific biomes highlighted overlap of the migrant SSL but not the deeper layers (Fig. 3, A and B).

Given that daytime foraging dives by the predator almost never overlapped with the deeper scattering layers (i.e., the nonmigrant DSL in the California Current and both DSLs in the North Central Pacific), we estimated the energetics of these hypothetical dives and contrasted them against the energetics of the observed dives that overlapped with the targeted scattering layer within each biome. For example, in the northern California Current, a foraging dive to the nonmigrant DSL (∼400 m) might result in a metabolic rate 5% higher and overall cost 56% higher than a behaviorally comparable dive to the migrant DSL (∼200 m). Alternatively, a dive to the nonmigrant DSL that is equivalent in overall cost to a shallower dive that overlaps with the migrant DSL would necessitate a 75% decrease in dive duration at foraging depths. In the North Central Pacific, foraging dives to the migrant (∼400 m) and nonmigrant (∼500 m) DSLs might result in a metabolic rate 20 or 31% higher and overall cost 80 or 121% higher, respectively, than a behaviorally comparable dive to the migrant SSL (∼200 m). Alternatively, a dive to the migrant DSL that is equivalent in overall cost to a shallower dive that overlaps with the migrant SSL would necessitate an 80% decrease in dive duration at foraging depths. A dive to the nonmigrant DSL cannot be made energetically equivalent to a migrant SSL dive solely via reduction in duration at foraging depths.

## DISCUSSION

Deep-diving albacore exhibited daytime overlap with scattering layers throughout the North Pacific. Biome-level variation in temperature, light attenuation, and scattering layer structure modulated the bioenergetics of this behavior as a tradeoff between foraging dive duration and metabolic rate. These results suggest that deep diving helps physiologically capable pelagic predators to achieve bioenergetic balance as part of a broader repertoire of foraging strategies. The importance of this mesopelagic strategy relative to epipelagic ones changes across biomes in concert with variation in their relative energetic profitability. Albacore diet in the California Current is largely constituted by epipelagic forage fish, with a comparatively smaller but substantive proportion composed of vertically migrating mesopelagic prey (*29*, *30*, *31*). In contrast, North Pacific albacore diet is increasingly composed of mesopelagic prey farther offshore (*32*, *33*) and in more tropical water (*34*). This greater reliance on mesopelagic prey is paralleled by warmer minimum temperatures experienced during foraging dives to the scattering layer that reduce the metabolic rate and extend the duration of such behavior. However, this biome-level juxtaposition is seemingly enabled by the addition of an SSL in the oligotrophic subtropical gyre and transition zone that, if not present, would otherwise necessitate dives into markedly deeper, colder water to reach the DSLs and thereby inflate the metabolic rate and overall cost of scattering layer forays in the open ocean. The shift in albacore diet between more productive, neritic biomes to oligotrophic, offshore biomes mirrors a concurrent change in overall vertical distribution where albacore increase their daytime occupation of deeper strata (and largely do not approach the near-surface) where warmer temperatures at depth occur in other ocean basins (*35*, *36*, *37*). Similarly, the ratio of mesopelagic to epipelagic prey in the diet of albacore is higher in more oligotrophic/tropical than mesotrophic/temperate waters in various parts of the world ocean (*37*, *38*, *39*), suggesting that these bioenergetic results can be generalized as a model for understanding deep diving behavior with respect to such gradients across the globe.

The composites of shipboard acoustics in the North Pacific Subtropical Gyre and Southern Transition Zone were consistent with previous acoustic characterizations of the North Central Pacific, which highlighted the stable presence of backscattering below 500 m from migrant and nonmigrant DSLs and the comparatively patchy occurrence of an SSL from 200 to 300 m during the daytime (*40*, *41*, *42*). Similarly, the composites from the California Current matched previous observations from other bioacoustics platforms, with pronounced daytime scattering in this region consisting of a migrant DSL typically between 180 and 300 m and nonmigrant DSL below 400 m (*43*, *44*, *45*). The shallower daytime distributions of the migrant and nonmigrant DSLs in the California Current compared to those offshore is consistent with mesopelagic organisms occupying a preferred range of light intensity and, thus, a position deeper in the water column when in a biome characterized by low light attenuation (*15*, *25*) such as the oligotrophic North Pacific. This responsiveness of mesopelagic organisms to subsurface light levels is evident even across (sub)mesoscale fronts within the California Current, where scattering layers are more vertically stratified on the offshore than inshore side of these features because of the presence of clearer waters farther offshore (*44*). The structuring effect of light on the migrant and nonmigrant DSLs is present although the community composition of these mesopelagic fauna is largely distinct between the North Pacific Subtropical Gyre/Transition Zone and California Current (*46*), with the most notable difference being the more consistent presence of the SSL offshore. The variable but more consistent presence of an SSL appears to characterize subtropical gyres, as studies from the North Pacific (*41*), South Indian (*40*), and North Atlantic (*20*) all found a temporally patchy SSL in the corresponding subtropical gyre but the general disappearance of this feature in surrounding water masses. In areas typically devoid of an observable SSL, the seasonal appearance of this upper layer may represent the seasonal displacement and shifting boundaries of distinct water masses (*18*, *19*).

For diving pelagic predators, the characteristics of prey patches (including their vertical distribution) are far more important to foraging efficiency than overall prey biomass (*47*). The SSL in the North Central Pacific may be patchily distributed (*40*, *41*, *42*), but if this prey structure did not exist, then it would require albacore to dive to at least the top of the migrant DSL at depths >400 m (more than doubling the vertical distance travelled below the mixed layer) where temperatures approach the colder conditions experienced while deep diving in the northern California Current. The markedly reduced metabolic cost of targeting the SSL rather than (hypothetically) either the migrant or nonmigrant DSL in the North Central Pacific highlights the centrality of this patchy prey structure to balancing the energetic budget of deep-diving predators that are physiologically restricted to the upper mesopelagic. Furthermore, the orders-of-magnitude-higher light levels present at the daytime depths of an SSL than DSL (*15*) should (for a given volumetric density of prey) increase the rate of predator-prey encounters and, thus, food intake [sensu (*48*, *49*)].

Daytime targeting of the SSL at depth is not only a potentially viable strategy in comparison to targeting deeper scattering layers but may also be relevant when compared to the nighttime alternative of foraging on prey that migrate toward the surface. The duration of potential nighttime overlap of predators with mesopelagic prey in this oligotrophic biome is reduced by the earlier departure from, and later arrival to, the epipelagic by organisms undergoing diel vertical migration in subtropical gyres (up to 90 min before sunrise/after sunset) relative to the same migrations in waters with lower optical clarity (such as the California Current) where migration times are more closely coupled to sunrise/sunset (Fig. 4) (*50*). The temporal constriction of this nighttime foraging opportunity is exacerbated by a markedly lower migrating proportion of organisms in the central than eastern Pacific (*16*, *41*) that together may substantially increase the relative energetic profitability of daytime mesopelagic foraging by predators in the oligotrophic North Pacific. Among deep-diving species within the North Pacific Subtropical Gyre and/or Transition Zone, those that are more thermally limited appear to largely target depths consistent with the distribution of the SSL [e.g., albacore (this study) and blue shark (*51*)], while those with greater physiological scope increasingly target depths consistent with the distributions of the DSLs [e.g., swordfish (*52*) and northern elephant seal (*53*)].

The repeated migrations of albacore between the California Current and North Central Pacific highlight the regional predictability of prey in these two major foraging grounds. Albacore returning from the North Central Pacific in summer encounter the highest abundances of sardine, anchovy, and euphausiids in the northern California Current but the distributions of these prey exhibit substantial spatiotemporal variability (*54*–*56*). This is reflected in the flexible diet composition of albacore in the California Current from year to year (*31*). The abundance and horizontal distribution patterns of albacore prey in the North Central Pacific are not as well documented. Notably, the heat increment of feeding (a proxy for energy intake) measured in albacore occupying the North Pacific Subtropical Gyre and Southern Transition Zone is far higher than would be expected from the low availability of epipelagic prey (*57*). While the taxonomic composition of the SSL on which albacore appear to feed during the daytime in the North Central Pacific remains unknown, the mismatch between observed energy intake and epipelagic prey biomass is consistent with a primarily mesopelagic diet. The greater potential profitability of deep daytime foraging dives in the North Central Pacific (this study) may be facilitated by albacore primarily occupying this region during the wintertime deepening of the mixed layer (*58*). With North Pacific mixed layer depth at its winter maximum (*59*) and shoaling of isolumes during winter (*60*) resulting in a relatively shallower distribution of scattering layers (*61*), albacore may energetically benefit from reduced vertical distance between their thermal refuge and mesopelagic prey on seasonal time scales. The potential energetic value of these offshore biomes to deep-diving pelagic predators of the North Pacific is further corroborated by a global-scale model of blue shark habitat that predicted favorable upper mesopelagic foraging conditions in oligotrophic waters (*62*), satellite telemetry of diverse predators revealing high-use areas in the gyre and transition zone (*63*), and a fishery-dependent analysis that found aggregation of the predator community within oceanographic features hosting enhanced mesopelagic foraging opportunities in the gyre (*23*). The foraging of deep-diving predators in the subtropical gyre and transition zone biomes, even if only seasonally [e.g., white shark (*64*)] or during a particular life stage [e.g., salmon shark (*65*)], indicates that reliance on the prey resources of the open ocean is a major component of the ecology of many pelagic predators.

Given burgeoning interest in scattering layer fisheries (*66*), predictions for climate change to cause a global decline of scattering layer fauna (*67*), and scattering layer research being predominantly focused on the globally distributed primary DSL (*16*, *17*), increased effort is needed to understand the ecology and biogeography of the SSL in the mid-latitudes. The occurrence of an SSL in multiple subtropical gyres (*20*, *40*), the oligotrophic Red Sea (*68*), and elsewhere under specific oligotrophic conditions (*19*), merits investigation to determine whether high light penetration driving the DSL(s) deeper increases ecological niche availability via vertical segregation. Poor spatial and temporal sampling coverage across the world ocean precludes clear delineation of the core and seasonal distribution of the SSL, and it remains unknown why this prey structure has been observed as migrant in one subtropical gyre (this study) and nonmigrant in another (*20*). Such distribution and migration dynamics are critical to understanding the mechanistic links between oceanographic regime, scattering layer structure, and prey availability to predators that ultimately influence the ecosystem risks of extracting mesopelagic biomass (*69*). The presence of the SSL appears vital to achieving bioenergetic balance in oligotrophic waters for pelagic predators that are physiologically restricted to the upper mesopelagic. This understudied link appears to enable higher trophic level life in the open ocean, including supporting important global fisheries, and highlights the unquantified ecosystem services provided by the ocean twilight zone.

## METHODS

### Archival tagging and geolocation

All tagging was done in accordance with protocols approved by the National Oceanic and Atmospheric Administration (NOAA) Southwest Fisheries Science Center, Institutional Animal Care and Use Committee. The tagging dataset was generated by a collaborative effort between the NOAA Southwest Fisheries Science Center and the American Fishermen’s Research Foundation (*35*). Each albacore (*n* = 25) was fitted with one of three types of archival tag (Lotek LTD2310, Lotek LAT2810, and Wildlife Computers MK9) that recorded depth, ambient (water) temperature, internal (visceral) temperature, and relative light level at 30- or 60-s resolution. These fish were between 63.5- and 89.9-cm straight fork length at release, at large for 62 to 1034 (median, 345) days, and cumulatively travelled 279 to 37,005 (median, 5572) km horizontally. The most probable tracks of the albacore fitted with archival tags were constructed using a hidden Markov model comparing tag-based observations against oceanographic measurements and ocean-resolving model outputs to generate position estimates (see Supplementary Methods for more details on the tagging and geolocation).

### Dive extraction and classification

Daytime dives (after sunrise and before sunset) potentially in pursuit of scattering layer prey were extracted to examine behavioral variation and underlying environmental drivers across the biomes of the North Pacific. Albacore vertical movement behavior is closely coupled to the mixed layer (*35*, *36*); thus, we defined the start and end of individual dives as occurring 10 m below the mixed layer depth, facilitating quantitative comparison of dives across variable oceanographic conditions (see Supplementary Methods for detail on estimation of the mixed layer depth). Scattering layers occur at depths below the euphotic zone; therefore, we only included dives in which the maximum depth was greater than the lower depth limit of the DCM in each 10^o^ latitudinal band [≥mean DCM depth + 3 SDs (*70*)]. Dives were assigned three phases, with the bottom phase occurring at depths ≥75% of the max depth of the focal dive and descent and ascent phases occurring before and after the bottom phase, respectively, at depths <75% of the max depth of the focal dive. The dive duration was defined as the sum of the descent, bottom, and ascent phases. After filtering and multivariate classification of dives into distinct types, we retained U-shaped (sustained foraging) or V-shaped (prey searching) dives on 3064 (30%) of the 10,360 deployment days (see Supplementary Methods for details on dive filtering and classification).

### Bioenergetics

To quantify the bioenergetic costs of albacore diving to putatively forage on or search for scattering layer prey, we used a previously published generalized additive model predicting total metabolic rate (active + standard, mg O_2_ kg^−1^ hour^−1^) as a function of swimming speed (body lengths s^−1^) and water temperature (*71*). This model, based on experimental results from the closely related and physiologically similar Pacific bluefin tuna (*Thunnus orientalis*), yielded predictions for albacore at every time step of the archival depth-temperature series data from the tags (see Supplementary Methods for more details on the application of the bioenergetic model).

### Light attenuation profiling

Light attenuation profiles were constructed from the archival depth-light series data to characterize the subsurface optical environment experienced by albacore across North Pacific biomes. We expanded upon the trawllight package for R (*72*) to derive attenuation from tag-measured irradiance taken during albacore vertical movements (see Supplementary Methods for more details on the profiling methodology and compositing of clustered profiles). The overlap of albacore with scattering layers was assessed by analyzing filtered light attenuation profiles extending below the euphotic zone where chlorophyll is negligible and increased attenuation may represent the presence of high concentrations of scattering layer organisms (*73*). We developed a custom algorithm to detect marked increases in attenuation occurring deeper than the lower limit (99.7th percentile) of DCM depth distribution, thereby conservatively identifying the presence of scattering layers only where chlorophyll concentrations should not be confounding. The DCM depth distribution values, calculated per 10° latitudinal band, come from a global analysis of >500 biogeochemical Argo profiling floats yielding >68,000 vertical chlorophyll profiles (*70*). Our approach represents the first attempt to map predator overlap with putative scattering layers across their tracked distribution as derived from analysis of animal-borne measurement of depth-light series data.

### Among- and across-biome dive analyses

To compare dive metrics among biomes, we built linear mixed-effects models of the variables of interest as a function of modified Longhurst province. These models included a variance structure to account for heterogeneous variance among modified Longhurst provinces and a random effect for individual to account for the nested nature of the dataset. Across modified Longhurst provinces and dive types, the metabolic cost rate of diving was modeled as a power function of minimum temperature (see Supplementary Methods for details on province modification and related models).

### Bioacoustics composites

To assess biome-level variation in scattering layer depth and composition, we constructed depth-averaged, 24-hour composites of volumetric backscattering with acoustic Doppler current profile (ADCP) data from the Joint Archive for Shipboard ADCP (*74*) for three regions across our North Pacific study area: the North Central Pacific (26° to 40°N, 200° to 220°E), northern California Current (41° to 49°N, 228° to 235°E), and southern California Current (25° to 33°N, 239° to 246°E). The instruments included in the composites were Teledyne Research Instruments ADCPs (TRDI) with operating frequencies of 38 (8%), 75 (31%), and 150 (61%) kHz. This range of frequencies enabled detection of diverse organisms potentially contributing to pelagic predator diet; 38 and 75 kHz best detect (micro)nekton scatterers such as fishes and cephalopods, whereas 150 kHz best detects macrozooplankton scatterers such as euphausiids (*14*). The profiles included in the composites were determined as any 24-hour diurnal cycle for which the mean position of the cruise fell within one of the regions of interest: North Central Pacific (219 cruise days), northern California Current (305 cruise days), and southern California Current (226 cruise days) (see Supplementary Methods for more details on the acoustic methodology).

## Supplementary Materials

### This PDF file includes:

Supplementary Methods Tables S1 and S2 Figs. S1 and S2 References
