# Supplementary material for: A shallow scattering layer structures the energy seascape of an open ocean predator
Source: Sci Adv. 2023 Oct 4;9(40):eadi8200. doi: 10.1126/sciadv.adi8200 (PMC10550225; doi:10.1126/sciadv.adi8200)
Supplement: Supplementary file 1 — Supplementary Methods Tables S1 and S2 Figs. S1 and S2 References [file sciadv.adi8200_sm.pdf]

Science Advances
AAAS

Supplementary Materials for

A shallow scattering layer structures the energy seascape of an open ocean predator

Martin C. Arostegui *et al.*

Corresponding author: Martin C. Arostegui, martin.arostegui@whoi.edu

Sci. Adv. **9**, eadi8200 (2023)
DOI: 10.1126/sciadv.adi8200

This PDF file includes:

Supplementary Methods
Tables S1 and S2
Figs. S1 and S2
References

Supplement: A shallow scattering layer structures the energy seascape of an open ocean predator

Supplementary Methods

Archival Tagging and Geolocation

Albacore were tagged through the Albacore Archival Tagging Program, a collaborative effort between the NOAA Southwest Fisheries Science Center and the American Fishermen's Research Foundation (35; 75; 57). Between October 2001 and September 2015, 1086 albacore were released off the North American coast between Oregon and Baja California. The archival tag data of the 25 recovered individuals with deployments > 60 days were converted into a standardized eTUFF format to facilitate analyses (`tags2etuff` – (76)).

The most probable tracks of the albacore fitted with archival tags were constructed using the `HMMoce` package (77) for R (78). Observation-based likelihoods were derived from data collected by the tags, using four separate likelihood calculations: 1) An SST likelihood was generated for tag-based SST values compared to remotely-sensed SST from daily optimally-interpolated sea surface temperature (OI-SST, 0.25^o resolution) fields (79; 80); 2) Light-based likelihood was derived using estimates of longitude from each manufacturer's proprietary software (WC, GPE2; Lotek, LAT Viewer Studio); 3) Depth-temperature profiles recorded by the tags were used to calculate ocean heat content (OHC - (81)) compared to daily reanalysis model depth-temperature products from the HYbrid Coordinate Ocean Model (HYCOM, 0.08^o resolution; (82; 83)); 4) A "one-sided" bathymetry-based likelihood was calculated in which all areas where bathymetry (eTOPO, (84)) was $\geq$ tag-measured maximum daily depth were given a likelihood equal to 1. The resulting observation likelihoods (re-sampled to 0.25^o resolution), in all reasonable combinations, were convolved with a diffusive movement kernel that allowed swim speeds up to 2 m/s. For full details of the convolution, filtering, and smoothing components of the model, see (77). Parameter estimation of behavior state movement speeds and transition probabilities was performed using the Genetic Algorithm (85) and model selection of the best-fit combination of likelihoods used the Akaike Information Criterion (AIC). The mean of the resulting daily posterior distributions was used to calculate daily positions and, thus, a most probable track. Geolocation was conducted on Amazon Web Services and the Woods Hole Oceanographic Institution high-performance computing cluster, Poseidon. One fish, tagged as a 74.5 cm juvenile but recaptured as a 103 cm adult

(Table S1), had the adult portion of its data excluded from analysis due to a marked change in vertical behavior concurrent with its southerly movement through suspected tropical spawning grounds (86). Similarly, negligible data from the Kuroshio Current and Eastern Pacific Subarctic Gyres (Longhurst) provinces were excluded.

Dive Extraction and Classification

Dives were extracted with the `diveMove` package in R (87). The archival depth-temperature series data were used to generate daily mixed layer depth estimates (`RchivalTag` - (88)) – defined as the depth at which temperature is half a degree below sea surface temperature (89) – from which the centered 7-d rolling mean was taken as the daily mixed layer depth used in dive extraction. Extracted dives were then quality controlled by applying a series of filters to isolate semi-idealized individual dives with more clearly identifiable behaviors. This entailed excluding dives with a vertical sinuosity (90) < 0.75 in the descent and/or ascent phase, a descent and/or ascent rate < 0.1 m/s, and a non-continuous bottom phase. These filters helped to remove dive bouts (a series of dives) and artificially long individual dives attributable to narrowly missing the dive cutoff used in assigning the dive start and end, as well as dives with one or more phases interrupted by other behaviors.

Filtered dives were classified into distinct types via multivariate analysis. First, a principal components analysis (PCA) was conducted using descriptor variables including the proportions of each dive in the descent, bottom, and ascent phases, as well as the duration (in minutes) of each dive phase and the dive overall. Proportions were arcsine transformed and durations were square root transformed to improve the normality of skewed data distributions, and all data were scaled and centered in the PCA. We evaluated the statistical significance of principal component eigenvalues with a Monte Carlo randomization test. Second, the resulting scores of principal components with significant eigenvalues were then scaled, centered, and subjected to hierarchical cluster analysis using Euclidean distance and the true Ward clustering method (91). The optimal number of clusters was determined by varying the number of clusters and testing various clustering indices. The modal best number of clusters ($k = 4$) represented fundamental dive types exhibited by other pelagic predators including pinnipeds, seabirds, and sharks (92; 93); 1) U-shaped (or square) – high proportion of bottom phase, longer overall dive duration, likely representing sustained foraging at a target depth | 2) V-shaped – low proportion of bottom phase, shorter overall dive duration, likely representing prey searching | 3) Right-skewed – large proportion of ascent phase, intermediate overall dive duration, potentially a specialized behavior or combination thereof | 4) Left-skewed – large proportion of descent phase, intermediate overall dive duration, potentially a specialized behavior or combination thereof. We excluded right- and left-skewed

dives from further analysis due to their less frequent occurrence and unclear ecological function, focusing solely on U- and V-shaped dives.

Bioenergetics

Although the Pacific bluefin tuna differs from albacore in terms of its exact physiology (e.g., cardiac performance – (94)), it is the most closely related study species to albacore with sufficient published metabolic data available; both are among the tuna species that dive the deepest, or migrate furthest into temperate latitudes, and exhibit the greatest capacity for regional endothermy and physiological thermoregulation (95). In addition, both species occupy similar thermal preferences (96), and the size ranges of Pacific bluefin tuna used to develop the metabolic rate model (70 – 84 cm FL) were within the ranges of tagged albacore included in this study. Given that only vertical, and not horizontal, displacement is known at each time step during a dive, the swimming speed is solely estimated in the vertical dimension from the archival depth-time series and can sometimes be underestimated; thus, we calculated the mean of literature reported values for the minimum body lengths s^{-1} required by albacore to maintain hydrostatic equilibrium (97; 98) and reassigned the swimming speed of all time steps in which it was lower than this value to this minimum speed (0.62 body lengths s^{-1}). Although tunas are ram-ventilators and, thus, constantly in motion, observation of captive yellowfin tuna (*Thunnus albacares* – (99; 100)) and biologging of wild Atlantic bluefin tuna (*Thunnus thynnus* – (101)) revealed that tunas engage in gliding (i.e., are not actively beating their tail) during parts of dives and modify the ratio of active swimming to gliding depending on the phase of the dive. Thus, we also calculated the standard metabolic rate (i.e., that of a fish not actively swimming with an assumed active metabolic rate of zero) during dives using an equation parameterized for albacore bioenergetics (102) and then adjusted the GAM-predicted metabolic cost of each dive phase by proportions of active swimming to gliding unique to each dive phase (descent – 35% active:65% glide, bottom – 100% active:0% glide, ascent – 95% active:5% glide) to acquire the total metabolic cost of each dive. The specified proportions of active swimming to gliding for descent and ascent phases were within the range of kinematic values measured *in situ* on Atlantic bluefin tuna during dives (see Table 2 of (101)); for the bottom phase, we assumed 100% active swimming due to the reduced vertical movement (and, thereby, likelihood of gliding) in this phase and need to ram ventilate and maintain hydrostatic equilibrium in tunas. Daily fork length estimates were converted to daily whole weight estimates with a sex-pooled relationship for North Pacific albacore (103) for use in the standard metabolic rate calculations.

Hypothetical Dive Comparison

Daytime foraging dives below the euphotic zone in the mesotrophic California Current and oligotrophic North Central Pacific almost never overlapped with deeper scattering layers (i.e., the nonmigrant DSL in the former and the two DSLs in the latter), which precluded comparison against observed dives to the shallowest available layer in the different biomes (i.e., the migrant DSL in the former and the migrant SSL in the latter). Thus, we compared the energetics of hypothetical dives to the upper boundary of different scattering layers using climatological depth-temperature conditions from the World Ocean Atlas (104) within the contrasting regions from which we constructed ADCP composites. Namely, we compared dives to the migrant DSL (200 m) versus nonmigrant DSL (400 m) within the northern California Current and to the migrant SSL (200 m) versus migrant (400 m) and nonmigrant (500 m) DSLs in the North Central Pacific. In each of these two biomes, we parameterized hypothetical dives for an albacore of a given body size (75 cm fork length) with the biome-specific means of observed dive metrics and compared dives in terms of potential ecological trade-offs. First, we constructed behaviorally comparable dives that shared the observed mixed layer depth as the starting point, descent and ascent rates, and duration at foraging depth; however, the disparate target depths consistent with the different scattering layers entailed exposure to different temperatures, longer overall durations of the deeper dives due to increased vertical displacement, and thereby enabled calculation of the increase in metabolic rates and overall costs when diving to a deeper layer. Second, we constructed energetically comparable dives of the same overall cost that shared the aforementioned dive metrics except for duration at foraging depth; here, the reduction in duration at foraging depth needed to achieve energetic cost equivalency between a dive to a shallow versus deeper scattering layer can be interpreted as lost opportunity for predator-prey encounters. These alternative metrics for comparing hypothetical dives to different scattering layers are intended to bound the ecological reality of what could occur if the model predator conducted such dives; it is possible (and likely) that dives to deeper scattering layers might be neither behaviorally nor energetically identical to dives to shallower scattering layers due to dynamic ecological trade-offs governing this process.

Light Attenuation Profiling

Due to the coarse temporal resolution of the archival data, attenuation profiles were generated by compositing light measurements across 7-d rolling windows, discretizing the light measurements into 10-m depth bins, taking the geometric mean of each, and then calculating the attenuation between them; quality control required that input light profiles always decreased with depth and had no gaps larger than 20 m, and that output attenuation profiles were positive at all 1-m intervals. These profiles only used tag-based light

measurements from the 2-hr period around local solar noon (as determined by the daily geolocation positions); this minimized variation in ambient light caused by changes in the solar elevation angle relative to the ocean surface (73). The archival tags are largely restricted to detection of blue light irradiance with maximum spectral sensitivities of 465-470 nm (105; 106), which maximizes the depths over which light can be measured given the markedly restricted penetration of shorter (i.e., purple) and longer (e.g., red) wavelengths in the visible spectrum. We calculated the attenuation coefficient for tag-measured light with an equation (Eq. #3 – (73)) accounting for the manufacturer-specific number of units change per order of magnitude change in light level, D (Wildlife Computers – $D = 20$, (107); Lotek – $D = 32$, (108)). Profiles were further quality controlled by filtering out those with a mean absolute error $> 10\%$ of the corresponding tag manufacturer's D value.

To reveal the proxied vertical distribution of scattering layers and the concurrent optical conditions at shallower depths within each biome, filtered light attenuation profiles with algorithm-identified scattering layer overlap were classified into distinct types via multivariate analysis and composited. First, a principal coordinates analysis (PCoA) was conducted on a dissimilarity matrix, based on Gower's coefficient (109), calculated from the scaled and centered attenuation values within the depth range of 10-300 m (or shallower, depending on the biome); in this case, profiles were included that did not necessarily cover the full depth range. Second, the resulting scores of the principal coordinates were then scaled, centered, and subjected to hierarchical cluster analysis using Euclidean distance and the true Ward clustering method (91). The optimal number of clusters within each biome was determined by varying the number of clusters and testing various clustering indices. The modal best number of clusters (North Pacific Subtropical Gyre: k = 2, North Pacific Transition Zone: k = 2, northern California Current: k = 3, southern California Current: k = 3) represented within-biome variation in the vertical distribution of scattering layers and its structuring by light attenuation at shallower depths; average profiles were composited by cluster within each biome to display the primary patterns. Marine snow, or particles affecting water turbidity that could contribute to light attenuation, should not be confounding to the detection of scattering layers over the ecologically-relevant depth range of this study. Turbidity-driven light attenuation is at or near its minimum deeper than 150 m in the North Central Pacific (110) and deeper than 50 m – except near the seafloor on the continental shelf – in the California Current (111). Thus, although marine snow is present in low concentrations at scattering layer depths, the concentration of marine snow is negligibly changing at those depths and so is not contributing to the marked increases in light attenuation below the euphotic zone that we use to identify putative scattering layers.

Longhurst Province Modification and Dive Analyses

The Coastal California Current Province was divided into northern and southern halves at 35°N (referred to as the 'northern California Current' and 'southern California Current' herein), the data-scarce North Pacific Subtropical Gyre Province West was assigned to the North Pacific Tropical Gyre Province (referred to as the 'North Pacific Subtropical Gyre' herein), and the data-scarce Alaska Coastal Downwelling Province was assigned to the northern California Current. The North Pacific Polar Front Province (referred to as the 'North Pacific Transition Zone' herein) was not modified. Thus, the dive metric analyses were restricted to the northern and southern California Current, North Pacific Transition Zone, and North Pacific Subtropical Gyre. Metrics were modelled separately for each dive type (U and V) and included the total metabolic cost, metabolic cost rate, potential profitability, dive duration, bottom duration, maximum depth, mixed layer depth, temperature at mixed layer depth, minimum temperature, change in ambient temperature, change in internal temperature, rates of descent and ascent, change in relative light level, and mean light attenuation from 20 – 150 m. The model of daily cumulative vertical displacement also included the temporal resolution of the series data. Post-hoc pairwise comparisons among modified Longhurst provinces used the Tukey method. A caveat of assigning albacore track locations to statically defined provinces is that there is both seasonal and inter-annual variation in their boundaries; however, under dynamic boundary conditions, the majority of the study region is relatively stable in its province assignment except for the latitudinal extremes of the California Current and the area immediately to the west of the intersection of the northern/southern California Current with the North Pacific Transition Zone and Subtropical Gyre (see Fig. 6a in (112)).

ADCP Composites

Previous classifications of the global mesopelagic into biogeographic ecoregions (46) and biogeochemical provinces (113) both identified a North Central Pacific ecoregion/Subtropical Gyre province comprised of the North Pacific Subtropical Gyre and southern portion of the North Pacific Transition Zone, but disagreed over whether the California Current constituted a unique ecoregion/province due to its high faunal and environmental influence from the surrounding ecoregions/provinces. Thus, we focused on the North Central Pacific, northern California Current, and southern California Current as representative regions to assess their (dis)similarity in scattering layer structure. The 24-hr, depth-averaged composites were generated using ADCP backscattering data for cruises between 1990 and 2019 obtained from the Joint Archive for Shipboard ADCP (JASADCP; (74)). To compute total volumetric backscatter (Sv) from raw ADCP received signal strength (also known as echo intensity or Received Signal Strength Indicator, RSSI), we used the process described in (114) and (115). The instruments in the data were uncalibrated and, therefore, assumptions

for calibration parameters included in the calculation were made based on technical documentation and correspondence with the instrument manufacturer TRDI (via (116)). The units of Sv are dB, referenced to $4\pi{*}m^{-1}$.

Supplementary Results and Discussion

The metabolic cost rate (per unit time) predicted by the bioenergetics model is explicitly a function of the ambient water temperature and swimming speed of the fish. In the context of deep diving, this means that metabolic cost rate changes with depth due to the change in temperature from surface to deep waters, as well as any concurrent change in speed the fish might exhibit when going from the warm mixed layer to the cold waters below. For Pacific bluefin tuna (the regionally endothermic species on which the bioenergetic model was originally built - (71)), there is a U-shaped curve of metabolic rate vs ambient water temperature, with a thermal optimum at 15-20°C and higher cost rates occurring at higher and lower temperatures. Higher costs at lower temperatures (such as those experienced during deep dives) may be the result of metabolic and/or behavioral thermoregulation (117). In short, a way to maintain elevated body temperature at low ambient temperatures is by increasing tail-beat frequency, which increases the metabolic cost rate. This elevation of tail-beat frequency at temperatures below the thermal optimum is documented in both Pacific bluefin tuna (117) and albacore (118), which share the same highly advanced degree of regional endothermy (95) and thermal preferences (96). Consistent with this, we observed significantly faster descent rates by albacore in the northern (colder) than southern (warmer) California Current, and the North Pacific Transition Zone (colder) than North Pacific Subtropical Gyre (warmer), for both U and V dives (Table S2). Furthermore, the mean temperature at the mixed layer depth, to which albacore ascend after deep descents, was always observed as being in the range of 15.4–17.5°C (Table S2), which is within the known thermal preference of albacore ($\sim$ 15–20°C, (96)). This refutes the possibility that albacore in our study were diving deep for thermoregulatory purposes (i.e., to cool off from being in overly warm water) and, instead, directly supports that dives were vertically/temporally restricted by colder temperatures below optimum. This is consistent with thermal constraints typically modulating rather than motivating deep diving behavior for many marine predators (21).

Although the depth distribution of the migrant SSL in the North Central Pacific is similar to that of the migrant DSL in the California Current (Fig. 4), there are fundamental differences in the dynamics of these layers. Acoustic frequencies suggest different taxonomic compositions between the SSL and DSL(s) at locations in at least the North Atlantic (20) and North Pacific (42). While the DSL is spatially ubiquitous, the SSL is markedly patchier in its occurrence (40; 41). Similarly, in some regions, the SSL only appears

seasonally whereas the DSL is ubiquitous throughout the year (19; 119). Cruises that have transited the boundary between two biomes documented the sudden disappearance of the SSL but retention of the DSL in the second biome (20). Thus, the SSL, which occupies a markedly brighter optical environment (15), should not be considered an extension of the DSL.

Daytime acoustic scattering in the upper epipelagic of the North Central Pacific (Fig. 4a) suggests shallow daytime foraging as a possible strategy for albacore. While the exact composition of this near-surface scattering is unknown, it is likely comprised of a mix of larval fishes and mesozooplankton that do not represent viable prey for juvenile albacore. Sampling from the Northwestern Pacific documented larval myctophids, typically < 5 mm body length, distributed in the upper epipelagic during the daytime (120; 121). Stomach contents of nearly 1000 juvenile albacore from the North Pacific revealed the average size of consumed fish to be 6.19 cm, with the smallest consumed fish of any species being 7.3 mm (31). Similarly, surveys from the North Central Pacific documented an upper epipelagic mesozooplankton assemblage comprised primarily of < 1 mm copepods (122; 123). Previous diet studies of juvenile albacore found negligible copepods in gut contents (e.g., (124)). The limited evidence for ingestion of copepods by albacore suggests that they were secondarily ingested from primary prey items as they comprised a very low numerical proportion of the diet ($\leq 0.2\%$) and low frequency of occurrence ($\leq 0.5\%$) and are well below the size range of other targeted prey items. For these reasons, copepods are not considered part of juvenile albacore diet (30; 31). Thus, what scattering is present during the daytime in the upper epipelagic of the North Central Pacific largely appears to not represent viable forage for juvenile albacore. This further supports the greater relative energetic profitability of targeting the SSL at mesopelagic depths during the daytime.

Tables

Table S1: Summary of albacore tagged with internal archival tags in the North Pacific. S. Fork Len. = straight fork length (cm) at release and recapture. Tag Manu. = tag manufacturer (Wildlife Computers - WC). Time-at-liberty (TAL) is in days. Recovery latitude (Rec. Lat.) and longitude (Rec. Lon.) are the coordinates where the fish was recaptured. Distance (Cum. Dist.) is cumulative horizontal distance in km. Observation likelihoods (Geoloc. Obs.) indicate observations used in `HMMoce` to construct the most probable track: L=light-based longitude, B=bathymetry, S=sea surface temperature, O=integrated Ocean Heat Content.

Tag ID	Tag Date	Tag Lat. (°N)	Tag Lon. (°E)	S. Fork Len.	Sex	Tag Manu.	Tag Model	TAL	Rec. Lat. (°N)	Rec. Lon. (°E)	Cum. Dist. (km)	Geoloc. Obs.
394	2011-08-03	44.8	233.7	68.0|75.8	U	Lotek	LAT2810	378	46.8	234.8	11,067	LBO
396	2011-08-03	44.9	233.6	63.5|84.5	U	Lotek	LAT2810	652	35.2	145.2	22,188	LBO
1045	2006-08-06	46.0	235.1	80.0|92.0	U	Lotek	LAT2310	680	31.2	220.7	17,800	LBO
2381	2004-07-01	44.5	234.4	65.0|73.0	F	Lotek	LAT2310	427	45.5	234.0	7,398	LBO
2393	2004-06-30	44.7	234.2	66.0|90.0	U	Lotek	LAT2310	696	30.3	215.4	19,131	LBO
2398	2004-07-01	44.5	234.4	64.0|NA	U	Lotek	LAT2310	445	45.9	233.5	11,374	LBO
890	2015-09-19	47.0	234.9	69.5|78.0	U	Lotek	LAT2310	345	46.1	235.3	11,519	LBO
1246	2003-11-08	31.9	240.7	87.5|94.0	F	Lotek	LAT2310	293	30.9	242.3	1,495	LBS
1464	2006-08-06	46.0	235.0	75.0|88.9	M	Lotek	LAT2310	380	32.4	241.8	5,650	LBO
1967	2003-11-07	31.9	240.6	86.5|91.0	F	Lotek	LAT2310	294	30.9	242.3	1,894	LBO
1973	2003-11-07	31.9	240.1	89.9|97.0	M	Lotek	LAT2310	297	31.4	242.2	1,656	LBO
1974	2003-11-07	31.9	240.6	81.0|86.5	F	Lotek	LAT2310	273	31.4	242.2	986	LBO
1987	2003-11-08	31.9	240.6	88.0|NA	U	Lotek	LAT2310	287	31.8	241.8	4,448	LBO
1991	2003-11-08	31.9	240.6	87.8|92.5	M	Lotek	LAT2310	282	31.4	242.6	4,114	LBS
2082	2003-11-10	32.0	240.8	81.5|NA	U	Lotek	LAT2310	284	31.5	242.4	4,312	LBS
2088	2004-09-18	45.2	234.8	75.0|NA	U	Lotek	LAT2310	331	32.0	241.7	3,277	LBO
2605	2004-07-01	44.5	234.4	65.0|67.3	U	Lotek	LAT2310	62	45.8	233.8	279	LBO
2942	2004-08-12	31.2	242.3	82.5|89.2	F	Lotek	LAT2310	327	30.3	242.3	5,572	LBS
390167	2003-07-25	29.2	242.6	84.0|94.0	M	WC	TDR-Mk9	343	30.0	242.9	2,209	LBS
390173	2003-07-27	29.3	242.7	89.0|91.0	U	WC	TDR-Mk9	83	32.3	240.8	455	LBS
390191	2003-07-27	29.3	242.7	87.0|93.5	M	WC	TDR-Mk9	349	29.9	243.3	2,183	LBO
1090251	2011-08-03	44.8	233.7	65.0|NA	U	WC	TDR-Mk9	752	46.1	233.6	26,008	LBO
1090269	2011-08-04	44.9	233.7	64.5|NA	U	WC	TDR-Mk9	692	35.2	143.7	23,068	LBO
1190241	2011-10-08	46.5	235.0	74.5|103.0	U	WC	TDR-Mk9	1034	3.1	169.7	37,005	LBO
1490108	2015-09-20	47.0	234.9	75.0|NA	U	WC	TDR-Mk9	374	44.9	234.7	10,445	LBO

Table S2: Summary of U-shaped (sustained foraging) and V-shaped (prey searching) dive metrics by biome. For each metric, the biome-specific mean values [±95% confidence interval] resulting from linear mixed-effects models are shown alongside letters indicating the statistical significance (if different), or lack thereof (if shared), of post-hoc pairwise comparisons. Biome abbreviations: CCALN – northern California Current, CCALS - southern California Current, NPTZ – North Pacific Transition Zone, NPSG – North Pacific Subtropical Gyre.

	CCALN	CCALS	NPTZ	NPSG
U				
Total Metabolic Cost (mg O_2 kg^{-1})	92.4 [84.4, 100.4]b	84.8 [76.7, 92.8]b	117.3 [107.0, 127.5]a	87.7 [79.4, 95.9]b
Metabolic Cost Rate (mg O_2 kg^{-1} min^{-1})	5.69 [5.56, 5.82]a	4.35 [4.22, 4.49]c	4.97 [4.83, 5.11]b	4.2 [4.05, 4.34]c
Potential Profitability (min mg O_2^{-1} kg)	0.103 [0.098, 0.107]a	0.132 [0.128, 0.137]b	0.128 [0.123, 0.132]b	0.141 [0.136, 0.146]c
Dive Duration (min)	15.8 [14.5, 17.1]c	19.3 [18.0, 20.7]b	23.2 [21.4, 25.0]a	20.7 [19.2, 22.3]b
Bottom Duration (min)	9.8 [8.6, 11.0]a	12.4 [11.1, 13.7]b	16.3 [14.6, 18.1]c	12.9 [11.4, 14.3]b
Maximum Depth (m)	175 [167, 184]c	220 [211, 228]a	208 [200, 217]b	218 [210, 227]a
Mixed Layer Depth (m)	29 [23, 35]a	54 [48, 60]b	51 [45, 57]b	55 [48, 61]b
Temperature at Mixed Layer Depth (°C)	15.5 [15.1, 15.8]a	17.0 [16.7, 17.3]b	15.4 [15.1, 15.8]a	17.0 [16.7, 17.4]b
Minimum Temperature (°C)	7.40 [7.22, 7.59]c	9.27 [9.08, 9.47]b	9.47 [9.24, 9.70]b	10.69 [10.36, 11.02]a
Change in Ambient Temperature (°C)	-8.64 [-9.18, -8.10]a	-6.99 [-7.53, -6.44]b	-6.61 [-7.16, -6.06]bc	-6.21 [-6.77, -5.65]c
Change in Internal Temperature (°C)	-1.25 [-1.44, -1.07]a	-0.75 [-0.94, -0.57]b	-1.19 [-1.38, -0.99]a	-0.83 [-1.01, -0.64]b
Descent Rate (m s^{-1})	-1.01 [-1.09, -0.93]a	-0.81 [-0.89, -0.73]b	-1.01 [-1.10, -0.93]a	-0.71 [-0.79, -0.63]b
Ascent Rate (m s^{-1})	0.65 [0.61, 0.69]a	0.65 [0.61, 0.69]a	0.65 [0.6, 0.69]a	0.63 [0.59, 0.67]a
Change in Light (orders of magnitude)	-4.13 [-4.45, -3.80]a	-3.51 [-3.82, -3.20]b	-3.35 [-3.67, -3.04]b	-3.39 [-3.70, -3.07]b
Mean Attenuation 20–150 m (m^{-1})	0.041 [0.040, 0.042]a	0.040 [0.038, 0.041]a	0.037 [0.036, 0.039]b	0.035 [0.033, 0.036]c
V				
Total Metabolic Cost (mg O_2 kg^{-1})	41.2 [39.9, 42.6]a	33.7 [32.2, 35.1]c	36.2 [34.6, 37.7]b	27.4 [26.0, 28.7]d
Metabolic Cost Rate (mg O_2 kg^{-1} min^{-1})	5.02 [4.92, 5.13]a	4.05 [3.94, 4.16]c	4.21 [4.09, 4.33]b	3.34 [3.23, 3.45]d
Potential Profitability (min mg O_2^{-1} kg)	0.069 [0.066, 0.072]a	0.073 [0.070, 0.077]ab	0.075 [0.071, 0.078]b	0.075 [0.071, 0.080]b
Dive Duration (min)	8.06 [7.85, 8.27]b	8.35 [8.12, 8.57]ab	8.34 [8.10, 8.58]a	8.06 [7.84, 8.29]ab
Bottom Duration (min)	3.0 [2.8, 3.2]a	2.7 [1.5, 2.9]b	2.9 [2.6, 3.1]ab	2.2 [2.0, 2.4]c
Maximum Depth (m)	163 [158, 168]c	198 [193, 204]a	180 [174, 186]b	196 [191, 201]a
Mixed Layer Depth (m)	27 [22, 31]a	49 [44, 54]b	64 [59, 69]c	88 [83, 94]d
Temperature at Mixed Layer Depth (°C)	16.3 [15.9, 16.8]a	16.4 [15.9, 16.9]a	15.7 [15.2, 16.1]b	17.5 [17.0, 17.9]c
Minimum Temperature (°C)	7.73 [7.51, 7.95]d	9.5 [9.26, 9.73]c	10.49 [10.22, 10.75]b	12.71 [12.43, 12.99]a
Change in Ambient Temperature (°C)	-9.14 [-9.77, -8.50]a	-6.26 [-6.91, -5.62]b	-5.65 [-6.30, -5.01]c	-4.92 [-5.57, -4.28]d
Change in Internal Temperature (°C)	-0.43 [-0.50, -0.37]a	-0.26 [-0.33, -0.20]c	-0.32 [-0.39, -0.26]b	-0.19 [-0.26, -0.13]d
Descent Rate (m s^{-1})	-1.13 [-1.20, -1.06]a	-1.01 [-1.08, -0.94]b	-0.94 [-1.01, -0.86]b	-0.71 [-0.78, -0.63]c
Ascent Rate (m s^{-1})	0.76 [0.72, 0.81]a	0.68 [0.64, 0.73]b	0.60 [0.55, 0.65]c	0.53 [0.49, 0.58]d
Change in Light (orders of magnitude)	-3.94 [-4.25, -3.64]a	-3.35 [-3.66, -3.05]b	-2.66 [-2.96, -2.36]c	-2.32 [-2.62. -2.02]d
Mean Attenuation 20–150 m (m^{-1})	0.042 [0.040, 0.043]a	0.040 [0.039, 0.041]b	0.037 [0.036, 0.038]c	0.034 [0.033, 0.035]d

Figures

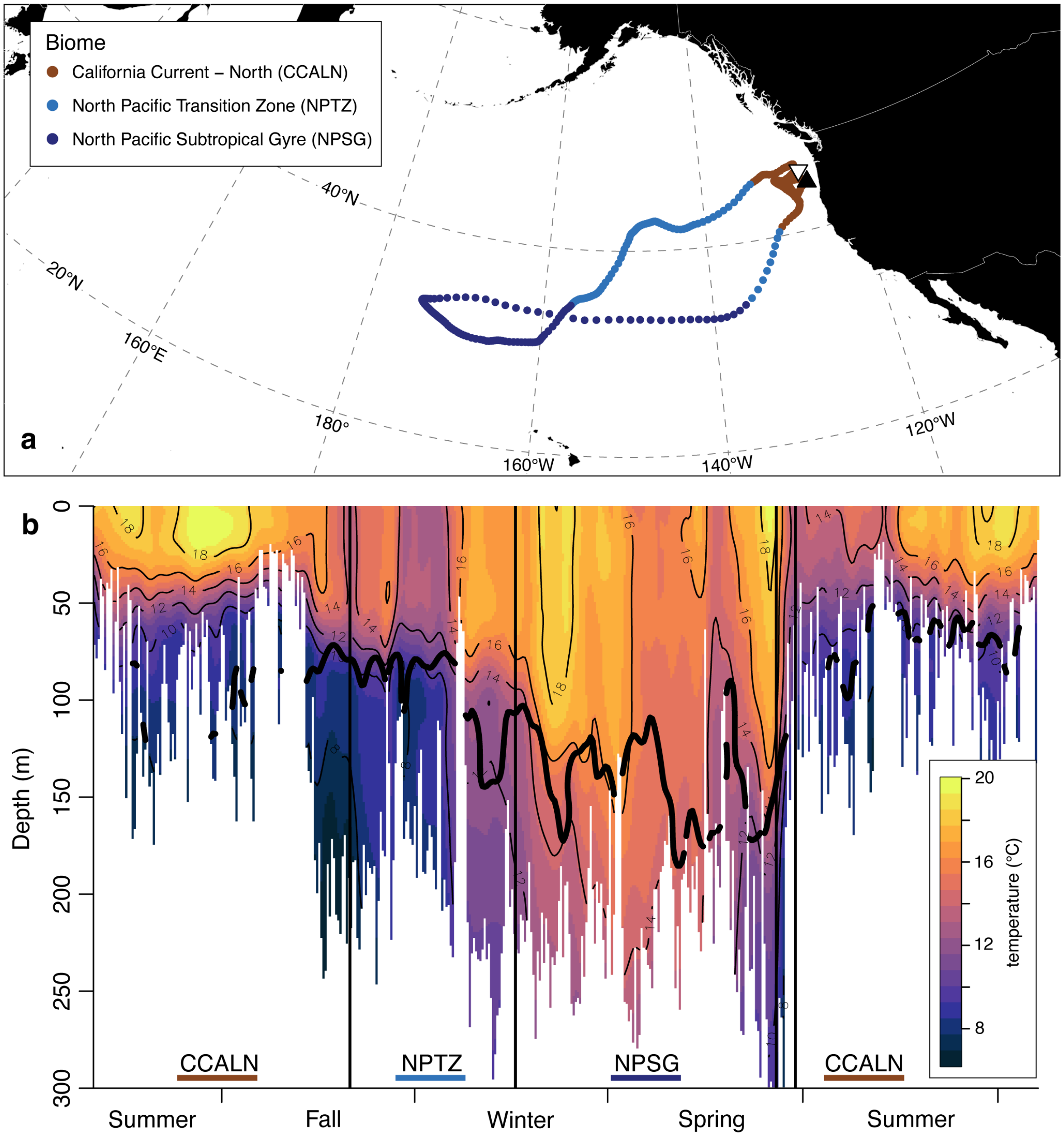

Figure S1: **Migration among oceanographic regimes.** Example seasonal migration of an individual albacore (Tag ID: 2398) across North Pacific biomes (a) and concurrent variation in the subsurface temperature and light conditions. In panel (a), the release and recapture points are indicated by the upward white and downward black triangles, respectively. In panel (b), isotherms every 2°C are shown as thin contour lines whereas a single isolume is shown as the thick contour line. CCALN – northern California Current, NPTZ – North Pacific Transition Zone, NPSG – North Pacific Subtropical Gyre.

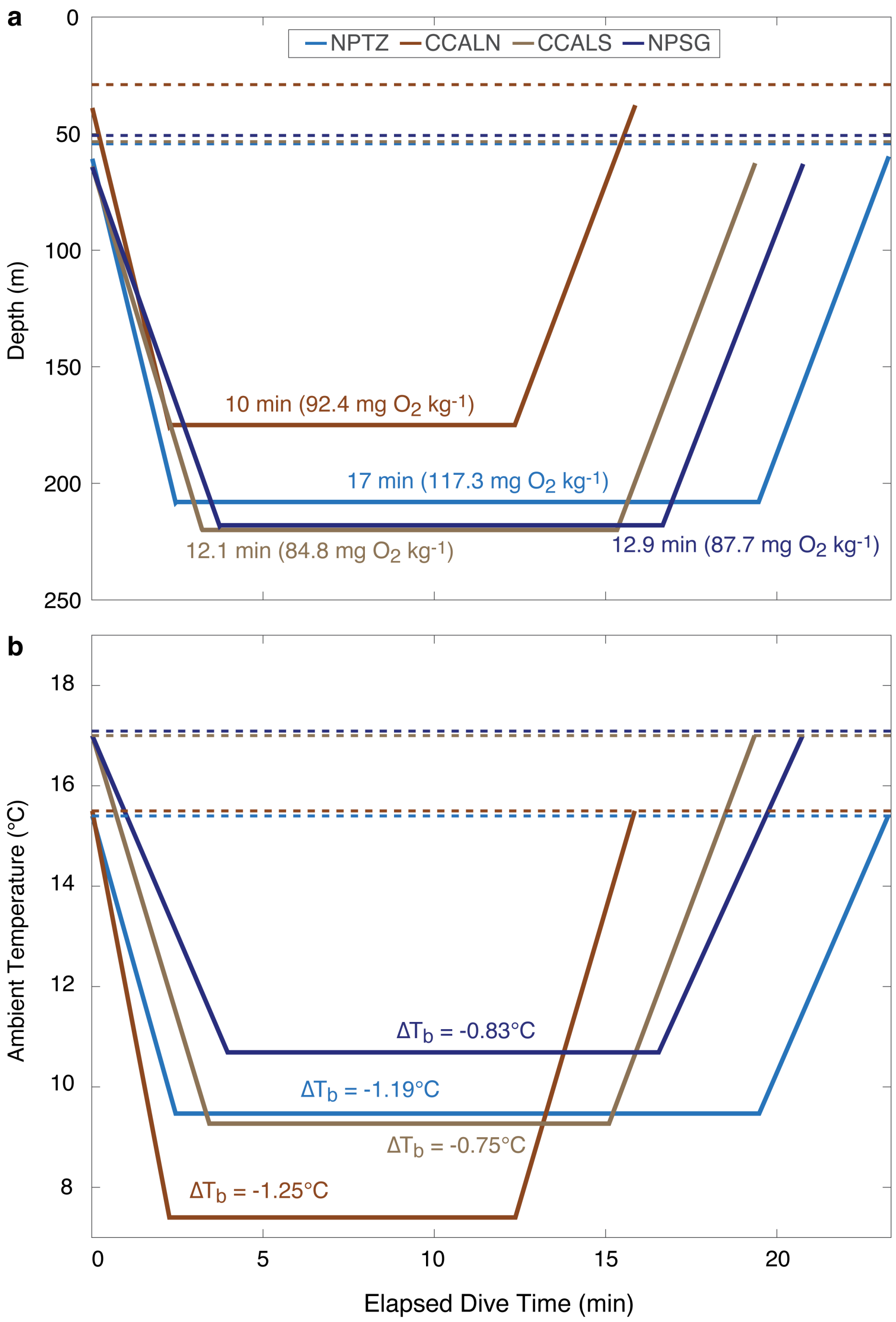

Figure S2: **Idealized deep daytime foraging dives.** Idealized U (sustained foraging) dives from each biome in terms of depth (a) and temperature (b). The biome-specific mean dive metrics (Table S2) were used to construct the mean idealized U dive from each biome. In panel (a), the dashed horizontal line represents the mean mixed layer depth in each biome, with dives starting and ending 10 m below the mixed layer; the mean bottom time (min) and mean total metabolic cost (mg O_2 kg^{-1}) of U dives are also provided per biome. In panel (b), the dashed horizontal line represents the mean temperature at the mixed layer depth in each biome; the mean change in internal temperature (°C) during U dives are also provided per biome.
